# Cannulation time is a more accurate measure of cannulation difficulty in endoscopic retrograde cholangiopancreatography than the number of attempts

**DOI:** 10.1093/gastro/got024

**Published:** 2013-08-24

**Authors:** Chenlu Tian, Anthony Gamboa, Biswashree Chaudhury, Field F. Willingham, Steve Keilin, Qiang Cai

**Affiliations:** Division of Digestive Diseases, Emory University, School of Medicine, Atlanta, GA

**Keywords:** endoscopic retrograde cholangiopancreatography, cannulation attempts, cannulation times

## Abstract

**Background**: Cannulation of the common bile duct (CBD) is the initial and sometime challenging step in endoscopic retrograde cholangiopancreatography (ERCP) procedure. Endoscopists often use cannulation attempts and cannulation time to grade cannulation difficulty, but a standard system has yet to be established. The objective of this study was to compare cannulation times with numbers of cannulation attempts, as measures of cannulation difficulty.

**Methods:** We conducted a prospective study in a tertiary referral center, enrolling 58 patients who were undergoing ERCP for a variety of indications. Cannulation time and the number of cannulation attempts were recorded for each patient. A subset of 14 ERCPs had two observers assessing attempts at cannulation. Cannulation time, number of attempts and inter-observer variability in assessment of attempts were compared and studied.

**Results:** The degree of agreement between two the methods (cannulation times and number of cannulation attempts) was unacceptable. There were considerable discrepancies between attempt tallies from two observers but the mean difference was statistically insignificant.

**Conclusion:** The grade of cannulation difficulty for a given ERCP procedure may differ when different methods are used (total cannulation time vs number of attempts); thus, grading by different methods should not be used interchangeably. Cannulation time is a more objective and more accurate assessment tool for grading cannulation difficulty than the number of attempts to cannulate the papilla.

## INTRODUCTION

Endoscopic retrograde cholangiopancreatography (ERCP) is an advanced endoscopic procedure that has been used in clinical practice for about four decades [1, 2]. It is commonly used for the diagnosis and treatment of biliary and pancreatic diseases [1–5]. Deep cannulation of the common bile duct (CBD) is the critical first step to visualizing the pancreaticobiliary system during ERCP procedure. Deep cannulation of the CBD at ERCP can represent a technical challenge, even to experienced pancreaticobiliary endoscopists [6–8]. In fact, the most common reason for an unsuccessful ERCP is the inability to cannulate the CBD [7, 8].

Previous studies have demonstrated that a difficult cannulation is a risk factor for post-ERCP complications, such as pancreatitis [8–10]. However, there has been no standardization of the assessment of cannulation difficulty. Methods of estimating difficulty have been variable and subjective, incorporating measurements that are difficult to define, such as the number of attempts to cannulate the papilla. In an effort towards a standardized system for grading cannulation difficulty, we undertook a comparative study evaluating accuracy of time taken to cannulate and the number of cannulation attempts.

## METHODS

### Patient enrolment

Seventy-two patients undergoing ERCP for a variety of indications were evaluated in this study, which took place from February of 2005 to August of 2006. Exclusion criteria included the following: pre-existing stent in CBD or pancreatic duct, history of endoscopic or surgical sphincterotomy, prior Billroth II surgery, Roux-en-Y gastric bypass surgery, ERCP within one week prior to the study and need for a biliary manometry study. All enrolled patients signed an informed consent document. The study was approved by our university's Institutional Review Board.

### Common bile duct cannulation

This study was carried out in a tertiary medical center where gastroenterology fellows are trained in ERCP. For each procedure, one of the ERCP fellows (a trainee) was the first endoscopist. If deep CBD cannulation was not achieved within 5 minutes, an attending (senior endoscopist) took the duodenoscope and continued the procedure. Two senior endoscopists took part in the study. Cannulation was performed using standard tapered or ball-tip ERCP catheters with 0.035 guide wires (Boston Scientific, Natick, MA, USA). In this study, the physicians were allowed to continue using their usual cannulation techniques. However, methods such as the pre-cut and pancreatic guide-wire or stent placement were not allowed.

Two methods of grading cannulation difficulty were assessed: cannulation time and number of cannulation attempts. Deep CBD cannulation time was defined as the time from starting cannulation to the time when the catheter had been introduced deeply inside the CBD, so that therapeutic procedures could be performed as needed. During the selective deep CBD cannulation process, if the pancreatic duct was cannulated, the endoscopist would immediately remove the catheter from the pancreatic duct and continue deep CBD cannulation. In this situation, the deep CBD cannulation time count was not interrupted. Deep CBD cannulation time count was also not interrupted when the attending endoscopist continued cannulation if the fellow failed to cannulate the CBD. Hence, the cannulation time recorded for the attempt included the initial time utilized by the fellow.

The number of cannulation attempts was tallied for each patient by an observer, who was an MD with two years of ERCP experience. For fourteen patients, an additional observer, who was an experienced pancreatobiliary endoscopist and had performed thousands of ERCPs, was brought in to separately record the number of cannulation attempts. Before the study, those two individuals were instructed on how to count the cannulation attempts. Each cannulation attempt was defined as the ERCP catheter—or the guide wire through the catheter—touching the major papilla, injecting/attempting to inject contrast or advancing/attempting to advance the guide wire through the ERCP catheter.

Cannulation failure was defined by the following criteria: the attending physician terminated the procedure, the major papilla could not be located or visualized in a suitable position and the patient became agitated and unsafe to complete the procedure. Failure was also recorded if cannulation time exceeded 30 minutes.

Two scoring systems were used in this study: time score and attempt score. A scoring system was used to grade cannulation difficulty, based on the amount of time elapsed and number of attempts: easy = total cannulation time of less than 5 minutes; moderate = total cannulation time of 5–10 minutes; difficult = total cannulation time greater than 10 minutes. Scoring based on number of attempts was defined as: easy = cannulation achieved in one attempt; moderate = cannulation achieved in two to five attempts; difficult = cannulation achieved in six or more attempts.

## STATISTICAL ANALYSIS

Comparison of total cannulation time and number of attempts, for evaluation of cannulation difficulty, was completed by calculating a Pearson correlation coefficient and performing the test of agreement between two clinical methods (comparing the z-score of measurements from both methods). *P*-value for significance was set at 0.05.

Student’s *t*-test was used to calculate agreement between two observers.

## RESULTS

Of the 72 patients referred for ERCP, 58 met the inclusion criteria and deep cannulation was successfully performed. Forty-four of these ERCPs were performed with a single observer recording the numbers of attempts. The mean cannulation time was 10.67 +/- 14.88 (Table 1). Cannulation was achieved in <5 min in 40% (23/58) and in >5 min but <10 min in 41% (24/58). The mean number of attempts was 8.34 +/- 12.45 counts (Table 1). The indications for ERCP in this study were common bile duct stones (*n=*30), bile duct stricture (*n=*21) and bile leak (*n=*7).

**Table 1.** Average cannulation time and attempt number

Scoring method	Mean	SD
Cannulation time*	10.7 minutes	14.9
Cannulation attempts**	8.3 counts	12.5

*n = 58 (single-observer and two-observer ERCPs)

**for 14 ERCPs with two observers, attempt number was recorded as the average of 2 observer counts

**Figure 1.**
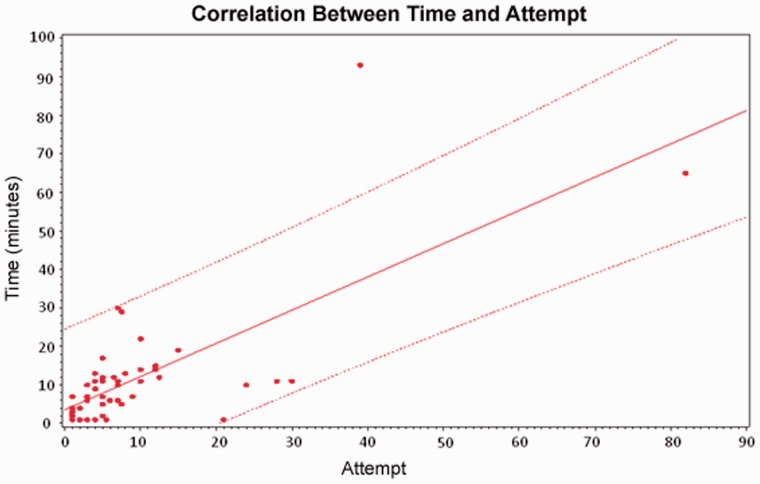
Time score derived from the following criteria: easy/1 = cannulation achieved within 5 minutes; moderate/2 = cannulation achieved in 5 to 10 minutes; difficult/3 = cannulation achieved in more than 10 minutes. Attempt score derived from the following criteria: easy/1 = cannulation achieved in 1 attempt; moderate/2 = cannulation achieved in 2 to 5 attempts; difficult/3 = cannulation achieved in 6 or more attempts.

**Figure 2.**
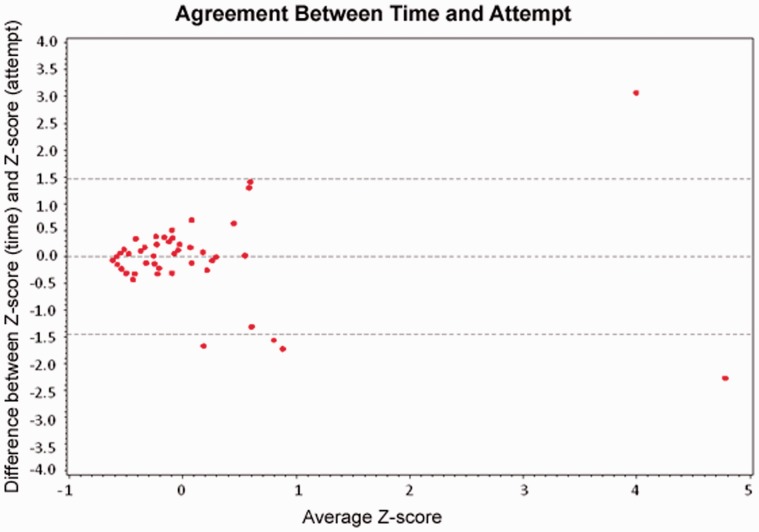
Time score derived from the following criteria: easy/1 = cannulation achieved within 5 minutes; moderate/2 = cannulation achieved in 5 to 10 minutes; difficult/3 = cannulation achieved in more than 10 minutes. Attempt score derived from the following criteria: easy/1 = cannulation achieved in 1 attempt; moderate/2 = cannulation achieved in 2 to 5 attempts; difficult/3 = cannulation achieved in 6 or more attempts. Difference obtained by subtracting the attempt score from the time score for each ERCP.

There was a positive correlation between cannulation time and the number of cannulation attempts; correlation coefficient 0.72 (Figure 1). However, the test for agreement between time and attempts showed at least five points that fall beyond the 95% confidence difference limits, making the degree of agreement between these two methods unacceptable (Figure 2).

**Figure 3.**
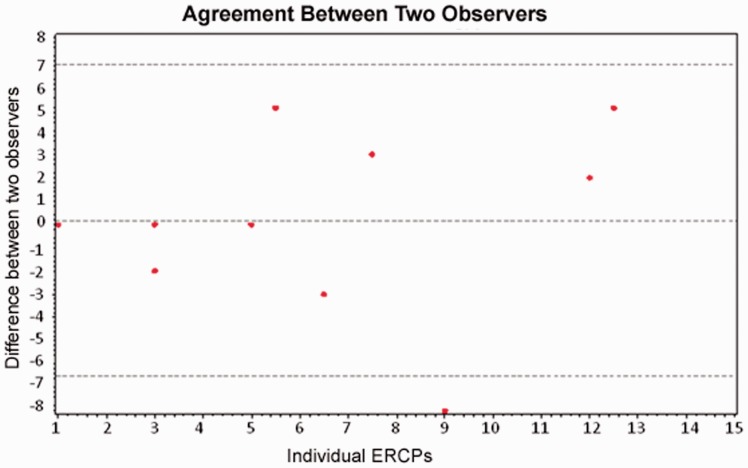
The count difference is the disparity in recorded attempts between two observers for each ERCP. For the 14^th^ ERCP, the count difference is not calculable as one observer lost count. The asterisk * denotes ERCPs where a count disparity placed the same procedure in different grades of cannulation difficulty. Cannulation difficulty graded by attempts is as follows: easy = cannulation achieved in 1 attempt, moderate = cannulation achieved in 2 to 5 attempts, difficult = cannulation achieved in 6 or more attempts.

Of the fourteen ERCPs with two observers separately recording numbers of attempts, the test of agreement between two observers resulted in a *P*-value of 0.797. However, the confidence intervals were wide (Figure 3).

## DISCUSSION

Endoscopic retrograde cholangiopancreatography remains an important therapeutic modality for pancreatobiliary diseases. Typically, the essential step in a successful procedure is cannulation of the CBD. The degree of difficulty during cannulation in ERCP is positively associated with post-ERCP pancreatitis [8]. Many studies have been published on the assessment of the degree of difficulty during cannulation for ERCP [9, 11–24]. Among those published papers, most of them used the number cannulation attempts as the measure of difficulty during cannulation [9, 11, 13–16]; three used cannulation time [12, 17, 18]; some used number of cannulation attempts together with cannulation time [19, 20] and a number of studies did not report the method of assessment of difficulty during cannulation [21–24]. However, there is no study comparing these two measures of cannulation difficulty. To our knowledge, this is the first study to compare these two assessments.

Data from this study show that, while there appears to be positive correlation between time and attempt for grading cannulation difficulty, the degree of agreement is unacceptable. Thus, grading of ERCP procedures based on different methods should not be used interchangeably. This finding further supports the need for standardization of measures of cannulation difficulty.

Although the numbers of cannulation attempts have been used by many authors, there is no uniform definition of a cannulation attempt. In one study, a cannulation attempt was defined by any repositioning or wedging of the catheter tip or cannulation device in an attempt to cannulate the biliary or pancreatic duct [14]; in another study, a cannulation attempt was defined as sustained contact between the cannulating device and the papilla for at least 5 sec [20]. Whilst the mean difference between attempt counts from two observers was not statistically significant in this study, the confidence intervals for limits of agreement were very wide, pointing to considerable discrepancies between counts from two observers. These differences were noted despite training and instruction in criteria for counting the cannulation attempts prior to initiation of the study. A larger study is needed to further elucidate the clinical significance of inter-observer variation.

It is commonly believed that a difficult cannulation is associated with increased post-ERCP pancreatitis. As the level of cannulation difficulty increased, so did the observed incidence of post-ERCP pancreatitis [8]. Since the aim of the present study was mainly to compare the numbers of cannulation attempts and the cannulation times, we did not follow up patients for post-ERCP pancreatitis or other procedure-related complications.

The main limitation of this study was that this was a single-center study.

Our results highlight a need for standardized grading of CBD cannulation difficulty. The presence of inter-observer variations—despite prior instruction on defining attempt counts—points to the subjectivity of this method. In contrast, cannulation time is more objective and not influenced by inter-observer variation. In some situations, such as when a patient is unstable during cannulation, the cannulation may be interrupted and may invalidate the cannulation time. However, based on our findings, we would recommend cannulation time as a more objective and thus superior method of grading cannulation difficulty.

## STUDY HIGHLIGHTS

- Previous studies have demonstrated that a difficult cannulation is a risk factor for post-ERCP complications such as pancreatitis [8–10].
- However, there has been no standardization of the assessment of cannulation difficulty. Methods of estimating difficulty have been variable and subjective, incorporating measurements that are difficult to define such as the number of attempts to cannulate the papilla.
- In an effort to achieve a standardized system for grading cannulation difficulty, we undertook a comparative study evaluating accuracy of cannulation time and the number of cannulation attempts.
- The grade of cannulation difficulty for a given ERCP procedure may differ when different methods are used (total cannulation time vs number of attempts); thus, grading by different methods should not be used interchangeably.
- Cannulation time is a more objective and more accurate assessment tool for grading cannulation difficulty than the number of attempts to cannulate the papilla.

**Conflict of interest:** none declared.
